# The auxotrophic *formate* (*for*) mutant of *Neurospora crassa* has significantly delayed growth but a normal circadian clock

**DOI:** 10.4148/1941-4765.2185

**Published:** 2024

**Authors:** Ziyan Wang, Kristin M. Lindgren, Jennifer J. Loros, Jay C. Dunlap

**Affiliations:** 1 Geisel School of Medicine at Dartmouth, Department of Molecular and Systems Biology, Hanover, NH, USA; 2 Geisel School of Medicine at Dartmouth, Department of Biochemistry and Cell Biology, Hanover, NH, USA

**Keywords:** frq, formate, luciferase, Neurospora, C24 mutation, single-cell assay

## Abstract

Some cell biological studies of *Neurospora crassa* have been limited by the rapid rates of hyphal growth and fusion. In this study, we investigated the causative mutation in the standard C24 allele of *for* (FGSC #9) and assayed the growth and circadian phenotype of the *for* strain under different nutritional conditions. We show that the *for* strain can be maintained as metabolically active single cells for 2 days before its growth advances into branched mycelia. This culturing system offers the potential to advance subcellular dynamic research and to facilitate greater understanding of *N. crassa* in the early developmental stages.

## Introduction

The filamentous fungus *Neurospora crassa* is a classical model organism for circadian research as well as other fields (Perkins and Davis 2000; Roche *et al*. 2014). During the asexual life cycle of *N. crassa,* single spores (conidia) form germ tubes within 4 hours when grown in adequate conditions and produce vegetative hyphae. Individual hyphae rapidly fuse through specialized conidial anastomosis tubes (CATs) to generate syncytia, interconnected hyphal networks, within 12 hours, thus allowing communication of temporal information (Davis 2000; Roca *et al*. 2005). It has been shown that gene expression profiles at different developmental stages in *N. crassa* colonies have huge variations (Kasuga and Glass 2008).

The molecular mechanism of eukaryotes’ central circadian clock is a transcription-translation negative feedback loop composed of positive and negative regulators (Dunlap 1999; Loros and Dunlap 2001). In *N. crassa*, the positive regulator White Collar Complex (WCC) is a heterodimeric transcription factor that drives the expression of *frequency* (*frq*), which encodes a main negative regulator FREQUENCY (FRQ), and various clock-controlled genes (*ccg*s) (Froehlich *et al*. 2002). FRQ, together with other components in the negative complex, represses the function of WCC (Froehlich *et al*. 2003). Such feedback loops, common to eukaryotes, take roughly 24 hours in accordance with the natural light-dark cycles on earth and provide huge selective advantages. Most traditional measuring methods for circadian clocks, including the race tube assay (Sargent *et al*. 1966; Belden *et al*. 2007), biochemical analysis (Nakashima 1981), and the later developed luciferase reporter assay (Gooch *et al*. 2008; Larrondo *et al*. 2015), only reflect the behavior of mycelial networks long after fusion between cells due to the time required for experimental set-up (Dunlap and Loros 2005). As a result, little is known about circadian behavior at the single cell stage when *N. crassa* is still growing as individual germlings that do not have physical contact with each other.

Maintaining *N. crassa* as metabolically active single cells for many hours is necessary to study the circadian behavior at early developmental stages before growth advances into branched mycelia and asexual differentiation. To achieve this goal, we examined several auxotrophic strains, including the formate requiring auxotrophic strain with a classic mutation *for* in the formate locus (NCU02274) (Lindgren 1994). The formate locus in *N. crassa* has been characterized (McClung *et al*. 1992). It encodes the highly conserved cytosolic serine hydroxymethyltransferase (SHMT), a pyridoxal phosphate (PLP) dependent enzyme that plays an essential role in cellular one-carbon pathways as well as amino acid biosynthesis. As this gene is essential, the knockout is only available as a heterokaryon (Colot *et al*. 2006). The C24 strain containing the *for* mutation generated by UV mutagenesis (Lein *et al*. 1948; Harrold and Fling 1952) has a deficiency in cytosolic SHMT enzyme activity and thus requires additional one-carbon sources for normal growth (Cossins *et al*. 1976; Cossins and Pang 1980; Jeong and Schirch 1996). However, the molecular nature of this formate requiring mutation has not been determined.

Here, we examined the molecular basis of the *for* mutation and determined, through microscopic assays, the detailed growth features of the *for* mutant strain. We find that the single cell stage can be maintained for days in basic medium without additional nutrient sources and that circadian clock function, including period length and temperature compensation, is normal in the *for* mutant strain. Thus, this strain can be used for circadian study in early-stage single cells.

## Results

### H223Y mutation is likely the reason for SHMT deficiency in the classical for mutant.

**Figure 1.**
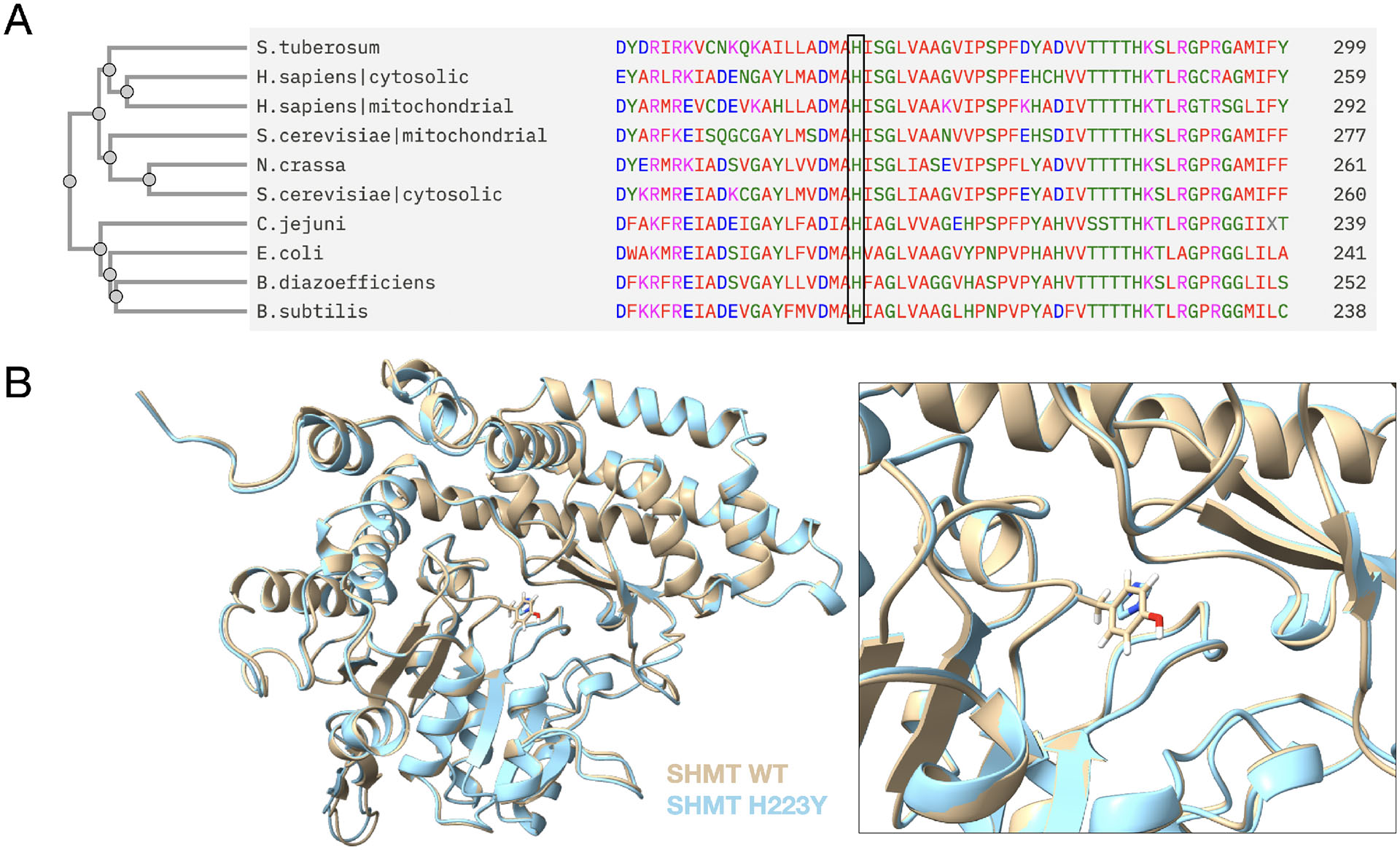
The canonical *for* allele carries a H223Y mutation in the PLP binding pocket of cytosolic serine hydroxymethyltransferase (SHMT). A) Amino acids sequence alignments of cytosolic or mitochondrial SHMT between *Neurospora crassa* and other organisms with the phylogenetic tree on the left. Conserved H223 is indicated by the black rectangle. B) Comparison between wild-type and mutated Alphafold-predicted structures of SHMT. Wild-type structure is shown in yellow. H223Y mutated structure is shown in blue.

To identify the underlying causative mutation in the standard C24 allele of *for* (FGSC #9), we sequenced the whole formate locus and surrounding sequences of a *for* containing strain and compared the results with wild-type sequence (McClung *et al*. 1992; Galagan *et al*. 2003). We identified a total of 12 mutations within the formate locus, with 3 in introns and 9 in exons (Supplementary Figure 1). The only nonsynonymous substitution was a C to T mutation leading to replacement of histidine with tyrosine at amino acid 223. Histidine 223 is highly conserved among both eukaryotes and prokaryotes (Figure 1A). Structure prediction based on *Neurospora crassa* SHMT sequence, along with solved structures from other organisms, indicates that this histidine is in the PLP binding pocket (Figure 1B) (Renwick *et al*. 1998; Scarsdale *et al*. 2000; Szebenyi *et al*. 2000). It is expected to form a hydrogen bond with the O3′ of PLP and interact with other amino acid residues at the active site to help catalysis. Mutating H230 in sheep SHMT (equivalent to H223 in *N. crassa*) to other amino acids, including to tyrosine, leads to an intense reduction in the enzyme catalytic activity (Talwar *et al*. 2000). Therefore, we believe the identified H223Y mutation is causative for the cytosolic SHMT deficiency, and the requirement for a one-carbon nutrient supplementation for growth of *for* mutant strains.

### The C24 for mutant has extremely limited growth while remaining viable for multiple days.

**Figure 2.**
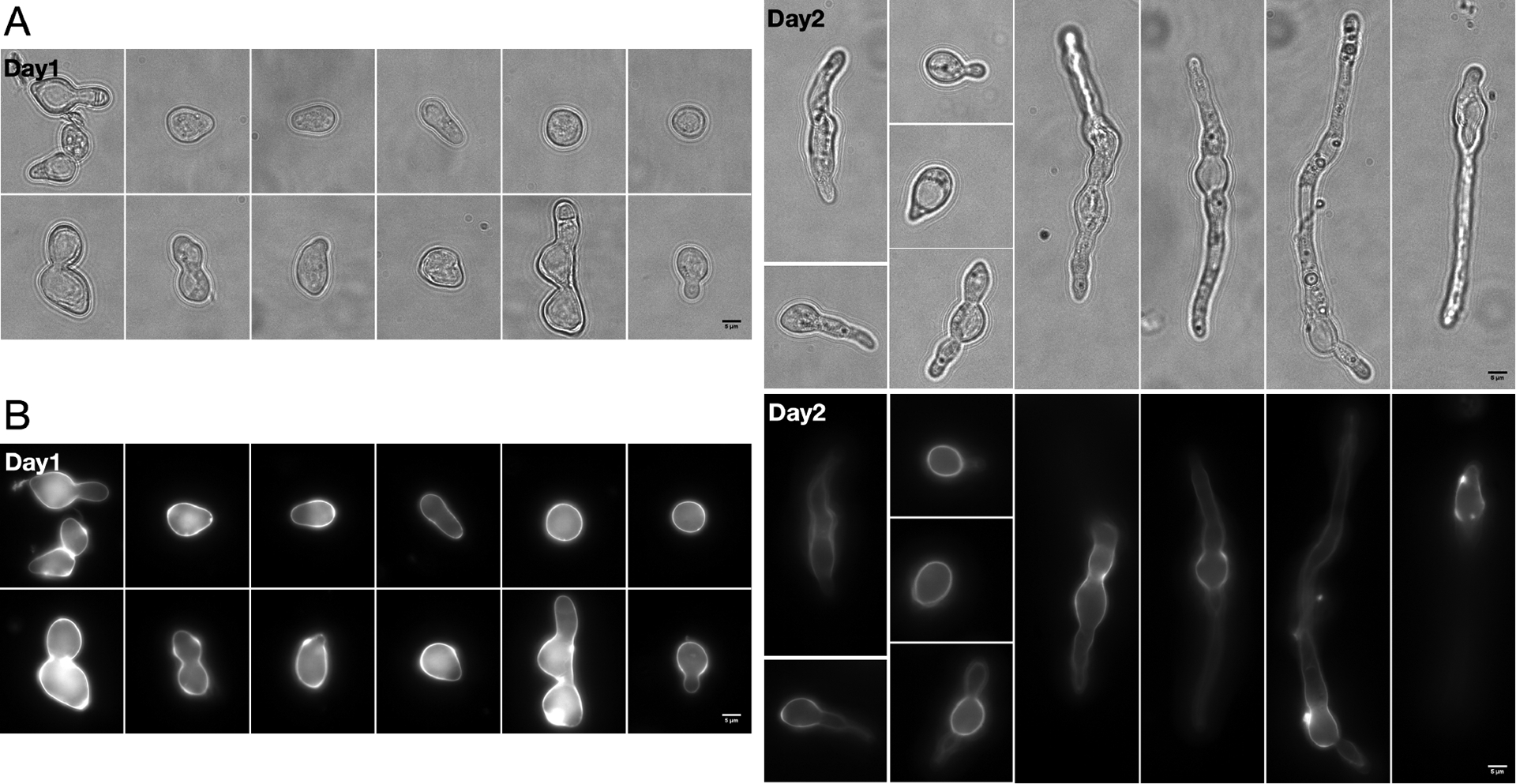
*for* conidia remain as germlings in liquid culture. Representative *for* cells (strain# 1949) after 1 or 2 days of growth at 25°C either A) under transmitted light or B) with CFW dye showing cell wall. Scale bar = 5μm.

*for* mutant strains require the addition of a one-carbon source like formate (CHO_2_^−^) for optimal growth (Harrold and Fling 1952). *N. crassa* can be maintained at the early stages of growth following germination, in which case they remain metabolically active but do not give rise to syncytial mycelia (Davis and de Serres 1970). We found that the C24 *for* mutant strain cultured in normal liquid medium (without formate or another additional one-carbon source) can stay viable at this single-cell stage for days without major visible growth. Within the first 2 days, microscopic examination reveals individual conidia with intact cell walls, and with or without very short germination tubes (Figure 2). There was little evidence of hyphal fusion or mycelial branching and cells maintained good viability (Lindgren 1994). Visible mycelial balls normally start to form on Day 3 in normal culture (data not shown), while the single-cell stage can be maintained for even longer with the addition of 0.1% Tween 80 in the culture medium (Lindgren 1994).

**Figure 3.**
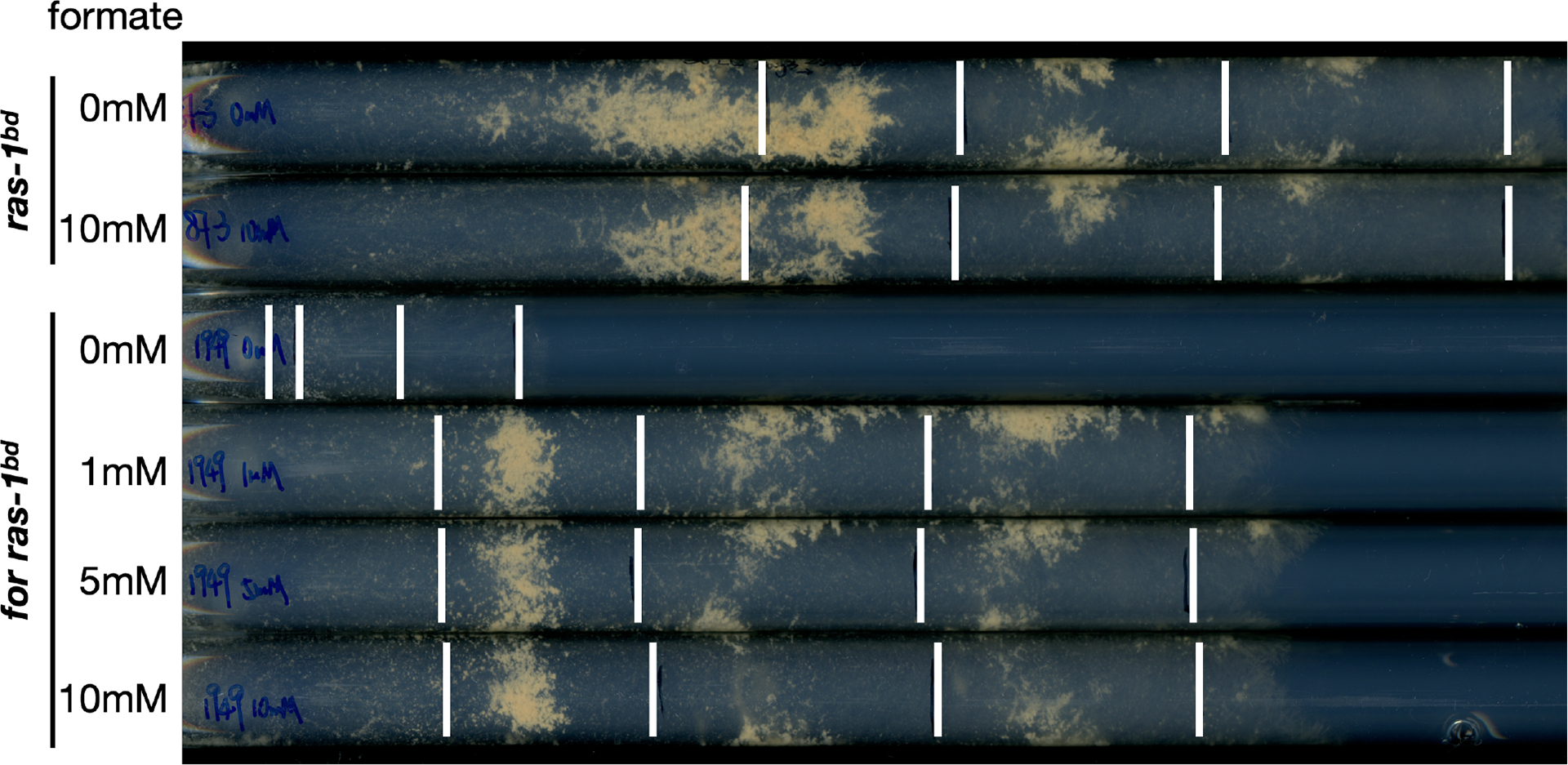
The *for* strain has a growth defect even with supplemented formate. Race tubes showing the growth rates of *ras-1*^*bd*^ (strain# 87–3) and *ras-1*^*bd*^; *for* (strain# 1949) strains under different concentrations of supplemented sodium formate. White bars mark the growth fronts every 24 hours staring from the end of day 2.

The *for* mutant strain also has a severe growth defect on normal solid medium without addition of a one-carbon supplement (0 mM formate), as shown on race tubes with the daily growth fronts marked (Figure 3). Consistent with liquid cultures, there was little visible growth in the first two days (before the first white bars). In addition to the deficiency in growth rate, the lack of conidiation when *for* was combined with the *ras-1*^*bd*^ genetic background indicated that asexual reproduction was also severely affected by the *for* mutation. With formate supplementation, the daily growth of the *for* mutant strain (*ras-1*^*bd*^*; for*) was rescued and comparable to the control (*ras-1*^*bd*^) in the later optimal growth stage. The control strain was not affected by formate supplementation as expected. Surprisingly, the onset of growth in the first two days is significantly slower for the *for* mutant strain even with formate in the medium. The growth phenotype did not change within the range of formate concentrations between 1 mM and 10 mM.

These results together highlight the importance of one-carbon metabolism in growth under different conditions, especially at earlier growth stages and during asexual reproduction. However, the *for* mutant can stay viable as single-cell germlings with minimal hyphal growth for multiple days without any one-carbon source added. This is likely due to the reduced but not completely absent SHMT activity in this mutant.

### The for strain displays normal circadian rhythmicity across formate supplementation levels and temperatures.

There are two common ways to test the circadian function in *Neurospora crassa*, one using the daily asexual reproduction banding pattern to examinate clock output, and the other using the luciferase reporter driven by the promoter of core clock gene *frq* to track the core clock (Dunlap and Loros 2005; Gooch *et al*. 2008). Although without supplementation the growth is too limited for the banding assay, circadian output of the *for* mutant is normal with the addition of various concentrations of formate (Figure 3). In addition, without supplemented formate, clock output was seen to be rhythmic by directly looking at the oscillation of the transcript of a clock-controlled gene *ccg-1* (Lindgren 1994).

**Figure 4.**
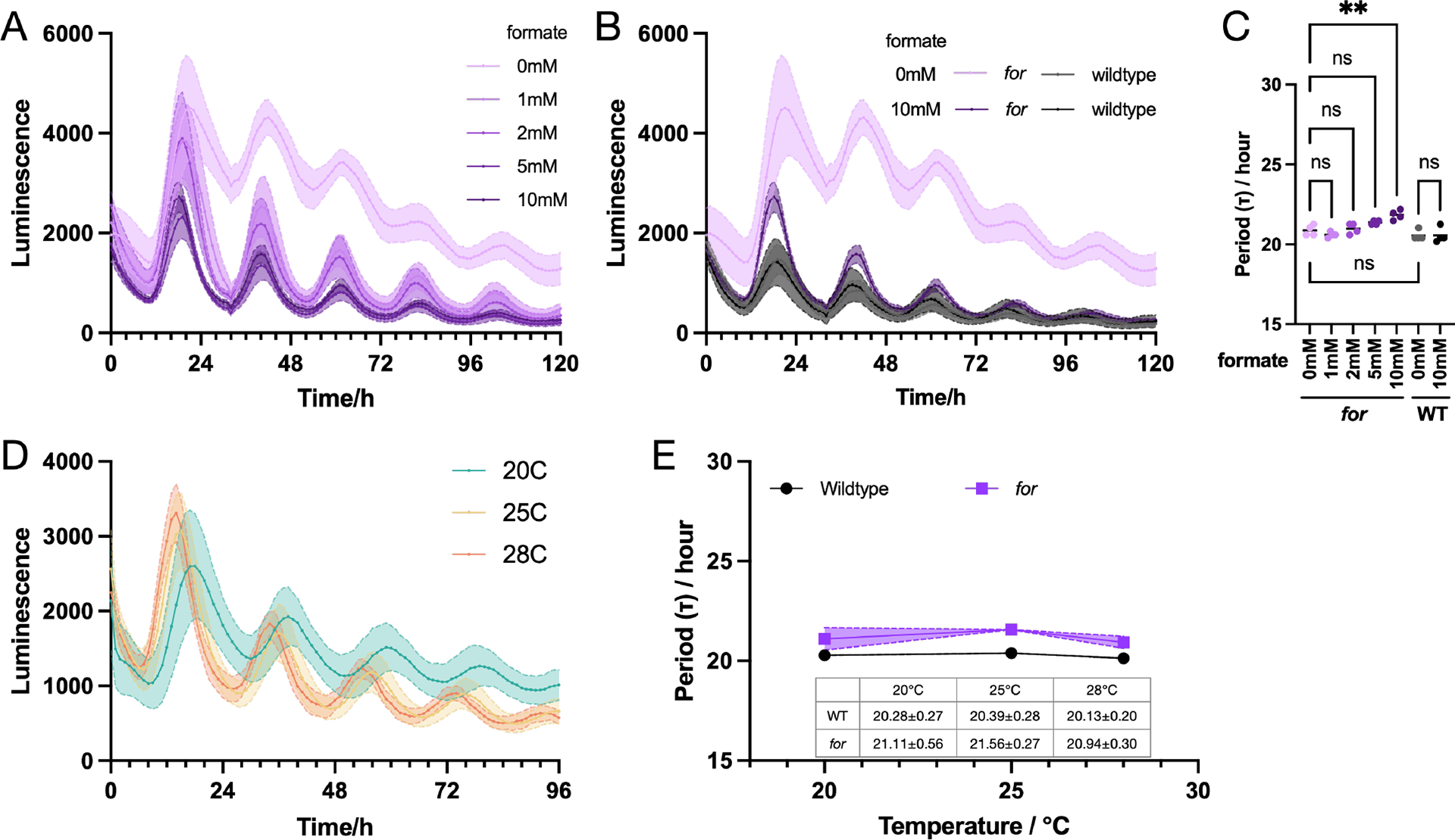
The *for* strain has a functional core circadian clock. A) Luciferase assays of the *for* strain at 25°C with different concentration of supplemented formate. B) Luciferase assays of wildtype at 25°C with or without supplemented formate. Each curve represents the average of 4 technical replicates with the shade as standard deviations. C) Period lengths of wildtype and *for-* strains across formate concentrations. D) Luciferase assays of the *for-* strain across temperatures with 10mM supplemented formate. E) Period lengths of wildtype and *for-* strains across temperatures. 3 biological replicates with 4 technical replicates each were performed for 3 selected temperatures; standard deviation is shown in the shaded region.

To examine the core clock activity, we monitored the activity of a *frq* promoter-driven luciferase reporter across formate levels and temperatures. The core clock activities were overall normal. In normal medium without additional one-carbon source (0 mM formate), the period length of the *for* mutant strain was the same as the wildtype control (Figure 4, A and B). There was marginal period elongation (< 1 hour) with increased formate concentration in the *for* mutant, while no difference was observed for the wildtype control (Figure 4 A–C). The circadian clock is still temperature compensated across physical environmental temperatures like the wildtype control (Figure 4, D and E).

Overall, the *for* mutation does not have major effects on the circadian clock in *N. crassa*. Additionally, the circadian clock is still detectable and functional in single cells in the absence of cellular fusion.

## Discussion

Deficient SHMT activity is seen in the classic *N. crassa* auxotrophic *for* mutant which bears a single animo acid mutation at the conserved histidine 223 in the PLP binding pocket. Conidia of this formate requiring mutant can be maintained as germinated single cells in minimal media and remain viable with little incidence of fusion for more than 48 hours. The circadian clock is overall normal in the *for* mutant cultured in medium with or without supplemented formate. These results confirm that the clock is functional in the earliest stages of the *N. crassa* life cycle. This research provided deeper understanding of a SMHT-deficient mutation and established the *for* mutant strain as a useful tool for future research of circadian behavior at the single cell stage.

As an intensively studied model organism, a significant number of *N. crassa* mutants arising from classical genetic screening are available although many of them have not yet been fully characterized (Baker 2009). We have determined the molecular nature of the *for* mutation. The associated formate locus has been characterized and known to encode the cytosolic SHMT (McClung *et al*. 1992). The only nonsynonymous substitution identified through our sequencing changes amino acid 223 from histidine to tyrosine. This histidine residue is highly conserved and has an important role in enzyme activity based on comparative and structural studies in multiple organisms (Figure 1) (Renwick *et al*. 1998; Scarsdale *et al*. 2000; Szebenyi *et al*. 2000; Talwar *et al*. 2000). This mutation is thus expected to have a reduced but not complete lost enzyme activity, which explains the formate auxotrophic phenotypes associated with *for* mutation and the difference compared to lethality of formate locus knockout.

In this study, we provide a method for culturing *N. crassa* as non-fusing cells in liquid culture for long periods of time. As a result of the partially defective SHMT in the *for* mutant, culturing in limiting nutrient conditions without an additional one-carbon supplement led to viable cells trapped in the germling stage instead of death (Figure 2) (Lindgren 1994). This feature makes the *for* mutant a great platform for studying some developmental processes at the unique single cell stage in the earliest development. That the central circadian clock is not affected in *for* mutant strain establishes a basis for using it in future circadian research (Figure 3, and 4).

One potential use of this culturing system is to advance subcellular dynamic research. Due to the fast vegetative growth rate of *N. crassa*, continuous recording of *in vivo* subcellular molecular behavior at high resolution for long periods (>2 circadian cycles, i.e. 48 hours) under microscope is challenging. Continuous imaging of a live vegetative *N. crassa* colony in previous research mostly lasted from several seconds to around 2 hours (Hickey *et al*. 2002; Hickey *et al*. 2004; Roca *et al*. 2005; Delgado-Álvarez *et al*. 2010; Roca *et al*. 2010), and longer monitoring requires the utilization of microfluidic devices with complex designs (Held *et al*. 2011; Lee *et al*. 2016; Cheong *et al*. 2024). The slow-growth single cell culturing system we describe provides an easy solution for recording whole cells at subcellular resolution over days. Other potential uses of this system in circadian clock research include implementation for a circadian clock mutant enrichment scheme, and evaluation of the potential for intercellular communication of phase information (Lindgren 1994)

## Materials and Methods

### Neurospora Strains.

**Table:** 

Lab Strain ID	Genotype	Mating type	Source or reference
661-5a	*his-3∷frq_cbox_-luc*	a	Larrondo *et al*. 2015a
87-3	*ras-1* ^*bd*^	A	Belden *et al*. 2007
837-211	*ras-1*^*bd*^ *; for*	A	Gift from Jerry Feldman
1949	*his-3∷frq_cbox_-luc; ras-1* ^*bd*^ *; for*	A	This study

Strain 1949 was generated through sexual crosses between 661-5a and 837-211,which contains the standard C24 allele of for from FGSC#9, using standard Neurospora methods (http://www.fgsc.net/Neurospora/NeurosporaProtocolGuide.htm).

### Sanger Sequencing and Multiple Sequences Alignments.

Genomic DNA of strain 837–211 was extracted using E.Z.N.A.^®^ Plant & Fungal DNA Kit (Omega Bio-tek). The whole *for* locus (NCU02274) was amplified by PCR using Phusion Flash High-Fidelity PCR Master Mix (Thermo Fisher), and then sequenced (3730 DNA analyzer, Applied Biosystems) using the following primers. The sequencing results were aligned to wildtype sequence in SnapGene.

**Table:** 

for seq 1	ctcggaggcgatgaggatg
for seq 2	ggaagagctgggcgttcttc
for seq 4	ccgtcagagacgagcttgtagc
for seq 5	cgtcgatgaggctgtcaagc
for seq 6	ggcgttgcaatttggtacttgg
for seq 8	ggcatcgtatctccagccct
for seq 9	gacccattggcatcgctgtt

Orthologs of SHMT included in the multiple sequences alignments were selected based on National Library of Medicine’s conserved domain database. Multiple sequences alignments were performed in Clustal Omega (Wang *et al*. 2023; Madeira *et al*. 2024 Apr 10).

### Structure Comparison.

Structures of the wildtype or mutated protein were predicted using AlphaFold2 (Jumper *et al*. 2021). The predicted structures were then imported into ChimeraX for further visualization and comparison.

### Microscopy.

*for* conidia from 7–10 day-old slant cultures were inoculated into 125-ml Erlenmeyer flasks with 50ml liquid medium (1× Vogel’s Salts, 0.1% glucose, 0.5% arginine, 50ng/ml biotin) and cultured at 110 rpm in constant light at 25°C for 3 days. 200 μl samples were removed from liquid culture every 24 hours, into a 1.5 ml microcentrifuge tube and mildly vortexed with 2 μl 5 mM calcofluor white (CFW; Biotium Item # 29067). After centrifugation in a microfuge at 900x g, 180 μl of the supernate was removed and the remaining 20 μl with tissue was used for microscopy. Transmitted-light and fluorescent images were taken immediately with a Nikon Ti-E widefield fluorescence microscope equipped with a Lumencor Sola Light Engine and an Andor Zyla 4.2 camera, using Chroma filter set 49028 (EX 395/25x and EM 460/50 m) and a 60X oil immersion objective. Images were processed in ImageJ.

### Race tube assay.

Conidial suspensions were inoculated at one end of the race tubes with race tube medium (1× Vogel’s Salts, 0.1% glucose, 0.17% arginine, 1.5% agar, 50 ng/ml biotin) supplemented with different concentrations of sodium formate (Thermo Scientific #141-53-7). Race tubes were cultured in constant light for 2 days before transferring to constant darkness at 25°C and growth fronts in each race tube were marked every 24 hours for 3–4 days as previously described (Dunlap and Loros 2005; Belden *et al*. 2007)

### Luciferase assay.

Conidial suspensions were inoculated into black 96-well plates containing race tube medium supplemented with 25 μM D-luciferin (GoldBio #115144-35-9) and sodium formate (Thermo Scientific #141-53-7), and were then covered by with a Breathe-Easy strip (USA Scientific). For formate concentration tests (Figure 4, A and B), inoculated plates were then entrained using 12:12 dark:light cycles for two days before being transferred to constant darkness for recording at 25°C. For temperature compensation tests, 25°C:20°C, 30°C:25°C, or 33°C:28°C 12:12 temperature cycles were used instead before recording at 20°C, 25°C, or 28°C, respectively. Luciferase signals were recorded using a CCD camera (Pixis 1024B, Princeton Instruments) for 15 minutes every hour for 5 days using LightField software (Princeton Instruments, 64-bit version 6.10.1). The mean intensity of each well was quantified and background-subtracted in ImageJ using a custom ImageJ Macro (Larrondo *et al*. 2015). Circadian periods were determined with a custom R program (Kelliher *et al*. 2020).

## Supplementary Material

1

## Data Availability

FGSC #9 can be obtained from the Fungal Genetics Stock Center. All strains used in this study and the original data (if not provided) are available upon request.
