# Supplementary material for: The auxotrophic formate (for) mutant of Neurospora crassa has significantly delayed growth but a normal circadian clock
Source: Fungal Genet Rep. Author manuscript; Available in PMC 2024 Nov 15. (PMC11565437; doi:10.4148/1941-4765.2185)
Supplement: 1 [file NIHMS2031133-supplement-1.pdf]

Supplementary Figure 1. Sanger sequencing results of the standard C24 allele of *for* aligned to wild-type sequence. The C to T nonsynonymous substitution is indicated by the red box.

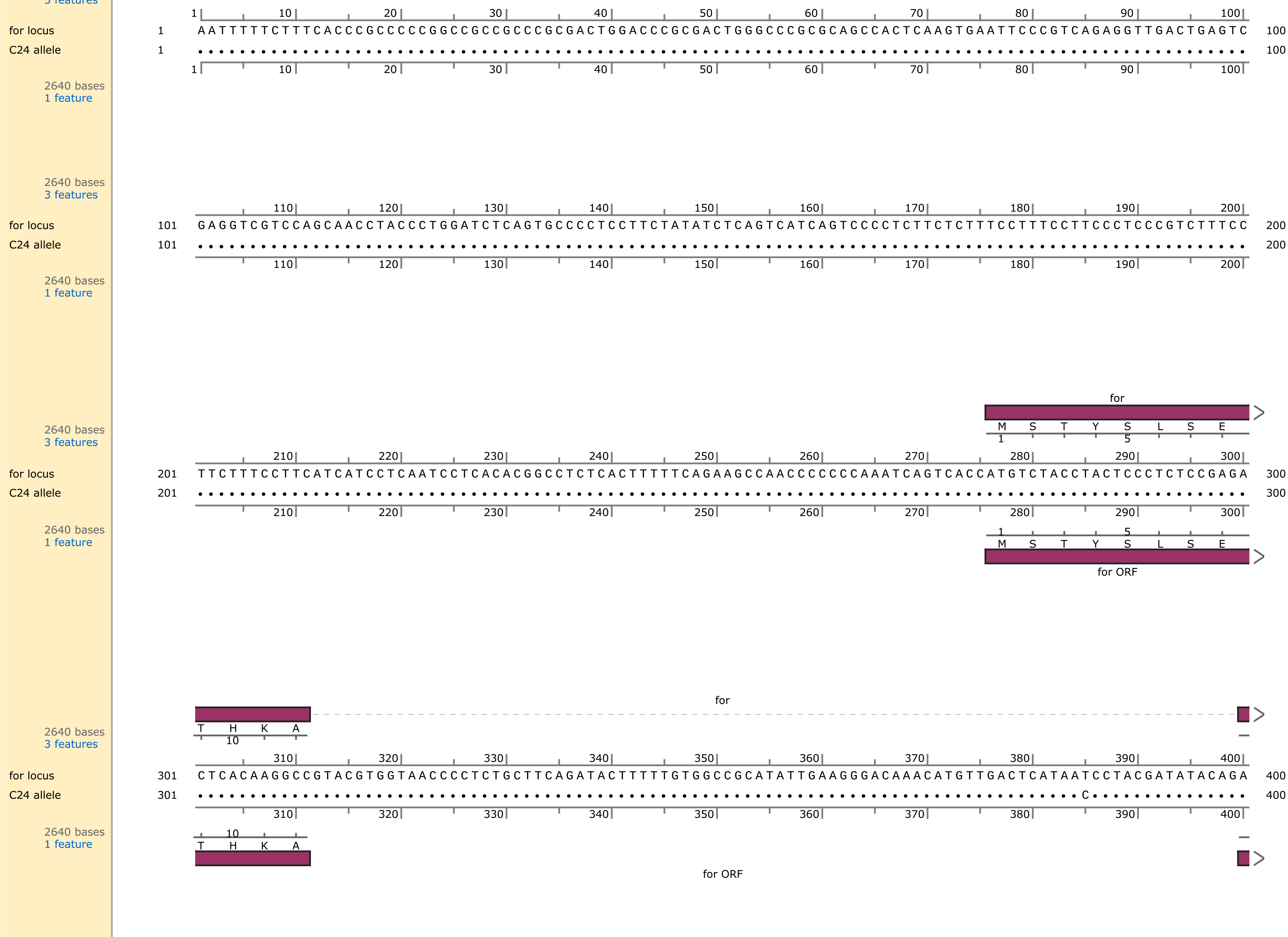

https://newprairiepress.org/fgr/vol68/iss1/1
DOI: 10.4148/1941-4765.2185

```
                for
2640 bases      M L E H S L V E S D P Q V A E I M K K E V Q R Q R E S I I L I A S E
3 features        15        20        25        30        35        40        45
for locus   401 TGCTCGAGCATAGCTTGGTCGAGTCCGACCCCCAGGTCGCCGAGATCATGAAGAAGGAGGTTCAGCGCCAGCGCGAGTCCATCATCCTCATCGCCTCCGA 500
C24 allele  401 .................................................................................................... 500
2640 bases        15        20        25        30        35        40        45
1 feature       M L E H S L V E S D P Q V A E I M K K E V Q R Q R E S I I L I A S E
                for ORF
```

```
                for
2640 bases      N V T S R A V F D A L G S P M S N K Y S E G L P G A R Y Y G G N Q
3 features            50        55        60        65        70        75
for locus   501 GAACGTCACCTCGCGTGCCGTCTTCGATGCCCTCGGCTCCCCCATGTCCAACAAGTACTCGGAGGGTCTTCCCGGCGCCCGCTACTATGGTGGCAACCAG 600
C24 allele  501 .................................................................................................... 600
2640 bases            50        55        60        65        70        75
1 feature       N V T S R A V F D A L G S P M S N K Y S E G L P G A R Y Y G G N Q
                for ORF
```

```
                for
2640 bases      H I D E I E V L C Q N R A L E A F H L D P K Q W G V N V Q C L S G
3 features      80        85        90        95        100       105       110
for locus   601 CACATCGACGAGATCGAGGTTCTCTGCCAGAACCGTGCCCTTGAGGCCTTCCACCTCGACCCCAAGCAGTGGGGTGTCAATGTTCAGTGCTTGTCCGGCA 700
C24 allele  601 .......................T........................................................C................... 700
2640 bases       80        85        90        95        100       105       110
1 feature       H I D E I E V L C Q N R A L E A F H L D P K Q W G V N V Q C L S G
                for ORF
```

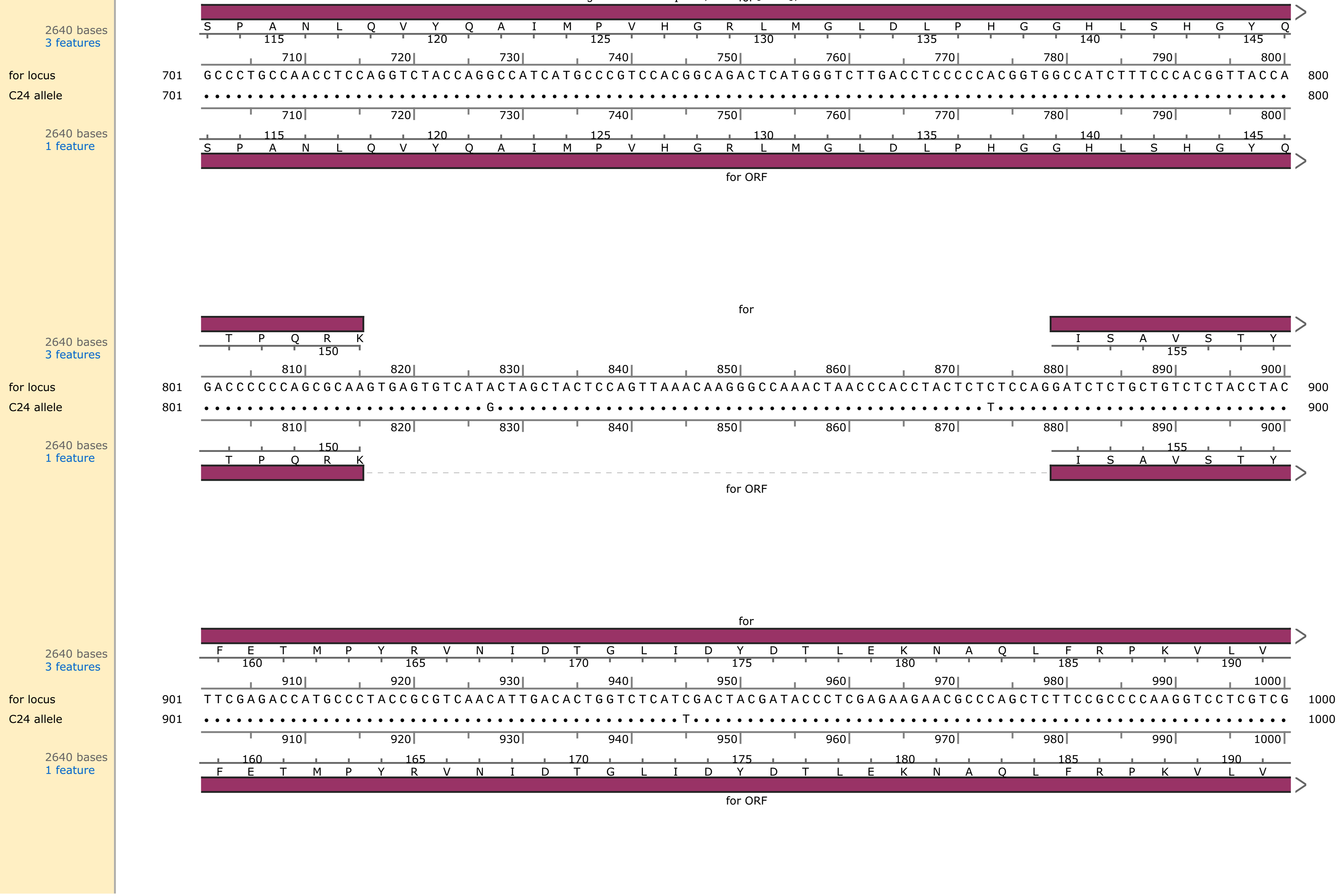
for
S P A N L Q V Y Q A I M P V H G R L M G L D L P H G G H L S H G Y Q
2640 bases
3 features
for locus 701 GCCCTGCCAACCTCCAGGTCTACCAGGCCATCATGCCCGTCCACGGCAGACTCATGGGTCTTGACCTCCCCCACGGTGGCCATCTTTCCCACGGTTACCA 800
C24 allele 701 •• 800
2640 bases
1 feature
for ORF
for
T P Q R K ... I S A V S T Y
2640 bases
3 features
for locus 801 GACCCCCCAGCGCAAGTGAGTGTCATACTAGCTACTCCAGTTAAACAAGGGCCAAACTAACCCACCTACTCTCTCCAGGATCTCTGCTGTCTCTACCTAC 900
C24 allele 801 ••••••••••••••••••••••••••G•••T••••••••••••••••••••••••••• 900
2640 bases
1 feature
for ORF
for
F E T M P Y R V N I D T G L I D Y D T L E K N A Q L F R P K V L V
2640 bases
3 features
for locus 901 TTCGAGACCATGCCCTACCGCGTCAACATTGACACTGGTCTCATCGACTACGATACCCTCGAGAAGAACGCCCAGCTCTTCCGCCCCAAGGTCCTCGTCG 1000
C24 allele 901 ••T••• 1000
2640 bases
1 feature
for ORF

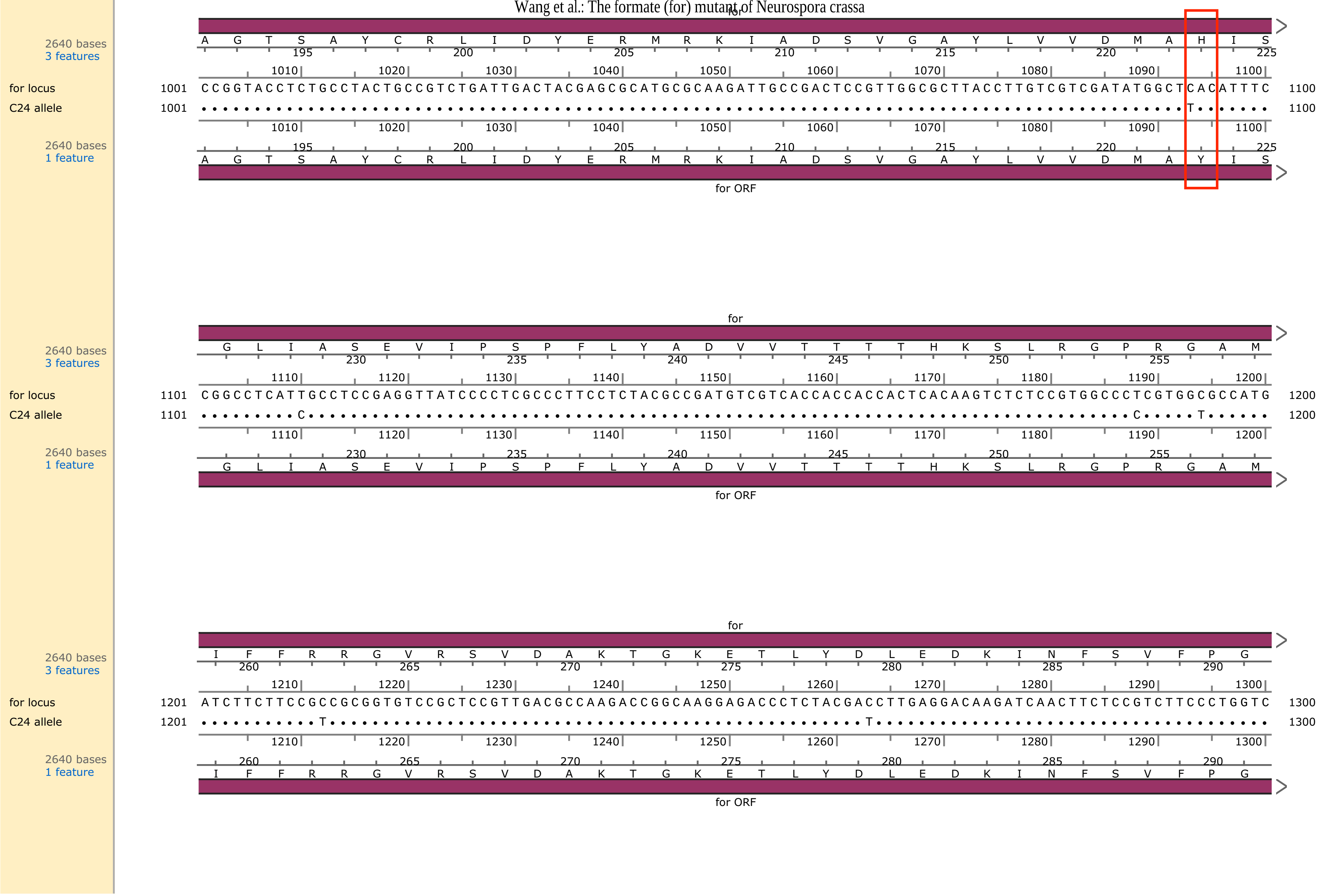
for
A G T S A Y C R L I D Y E R M K I A D S V G A Y L V V D M A H I S
2640 bases
3 features
for locus
1001 CCGGTACCTCTGCCTACTGCCGTCTGATTGACTACGAGCGCATGCGCAAGATTGCCGACTCCGTTGGCGCTTACCTTGTCGTCGATATGGCTCACATTTC 1100
C24 allele
1001 1100
2640 bases
1 feature
A G T S A Y C R L I D Y E R M K I A D S V G A Y L V V D M A Y I S
for ORF
for
G L I A S E V I P S P F L Y A D V V T T T T H K S L R G P R G A M
2640 bases
3 features
for locus
1101 CGGCCTCATTGCCTCCGAGGTTATCCCCTCGCCCTTCCTCTACGCCGATGTCGTCACCACCACCACTCACAAGTCTCTCCGTGGCCCTCGTGGCGCCATG 1200
C24 allele
1101 1200
2640 bases
1 feature
G L I A S E V I P S P F L Y A D V V T T T T H K S L R G P R G A M
for ORF
for
I F F R R G V R S V D A K T G K E T L Y D L E D K I N F S V F P G
2640 bases
3 features
for locus
1201 ATCTTCTTCCGCCGCGGTGTCCGCTCCGTTGACGCCAAGACCGGCAAGGAGACCCTCTACGACCTTGAGGACAAGATCAACTTCTCCGTCTTCCCTGGTC 1300
C24 allele
1201 1300
2640 bases
1 feature
I F F R R G V R S V D A K T G K E T L Y D L E D K I N F S V F P G
for ORF

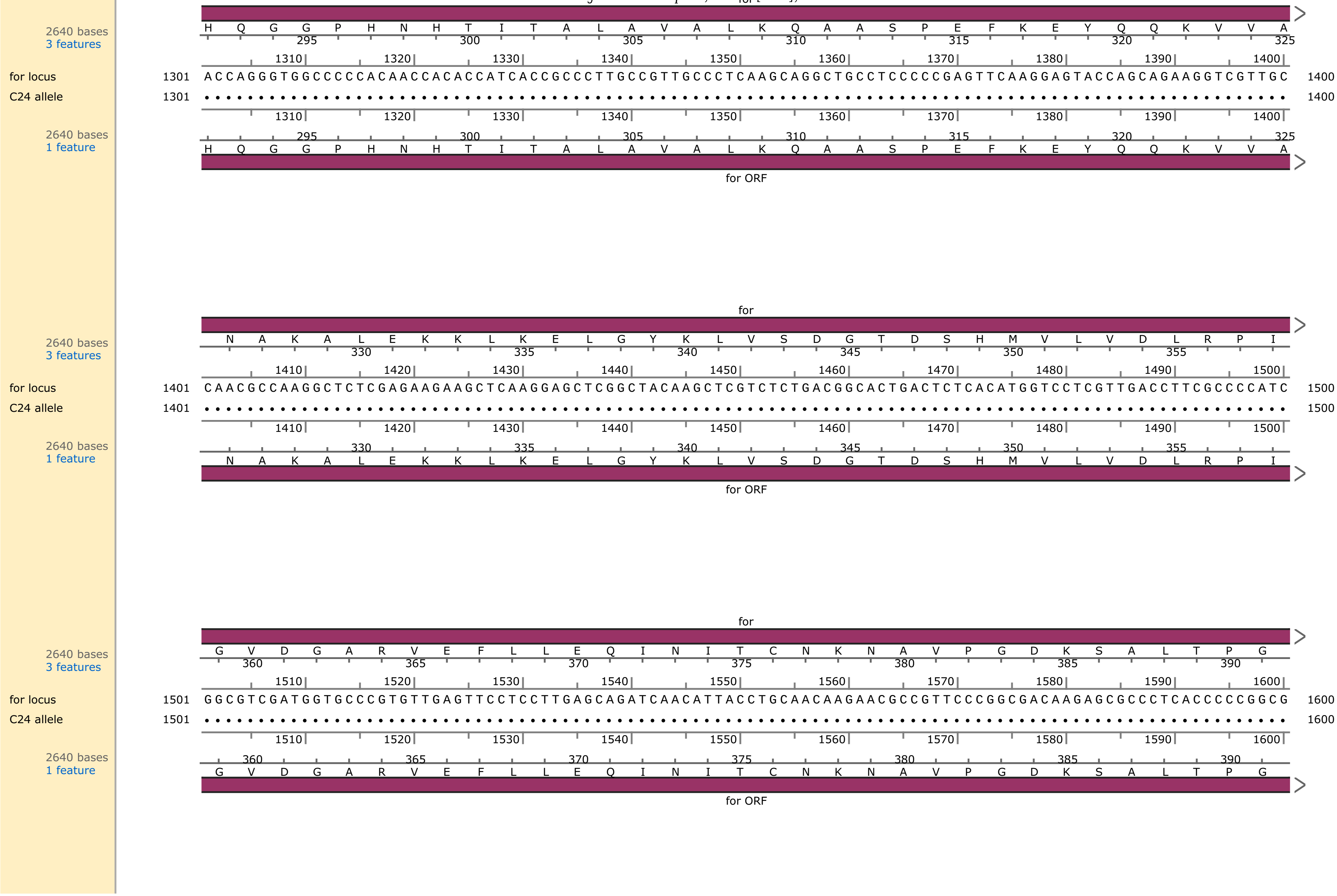

for
2640 bases
3 features
H Q G G P H N H T I T A L A V A L K Q A A S P E F K E Y Q Q K V V A
295 300 305 310 315 320 325
1310 1320 1330 1340 1350 1360 1370 1380 1390 1400
for locus 1301 ACCAGGGTGGCCCCCACAACCACACCATCACCGCCCTTGCCGTTGCCCTCAAGCAGGCTGCCTCCCCCGAGTTCAAGGAGTACCAGCAGAAGGTCGTTGC 1400
C24 allele 1301 1400
2640 bases
1 feature
for ORF
for
2640 bases
3 features
N A K A L E K K L K E L G Y K L V S D G T D S H M V L V D L R P I
330 335 340 345 350 355
1410 1420 1430 1440 1450 1460 1470 1480 1490 1500
for locus 1401 CAACGCCAAGGCTCTCGAGAAGAAGCTCAAGGAGCTCGGCTACAAGCTCGTCTCTGACGGCACTGACTCTCACATGGTCCTCGTTGACCTTCGCCCCATC 1500
C24 allele 1401 1500
2640 bases
1 feature
for ORF
for
2640 bases
3 features
G V D G A R V E F L L E Q I N I T C N K N A V P G D K S A L T P G
360 365 370 375 380 385 390
1510 1520 1530 1540 1550 1560 1570 1580 1590 1600
for locus 1501 GGCGTCGATGGTGCCCGTGTTGAGTTCCTCCTTGAGCAGATCAACATTACCTGCAACAAGAACGCCGTTCCCGGCGACAAGAGCGCCCTCACCCCCGGCG 1600
C24 allele 1501 1600
2640 bases
1 feature
for ORF

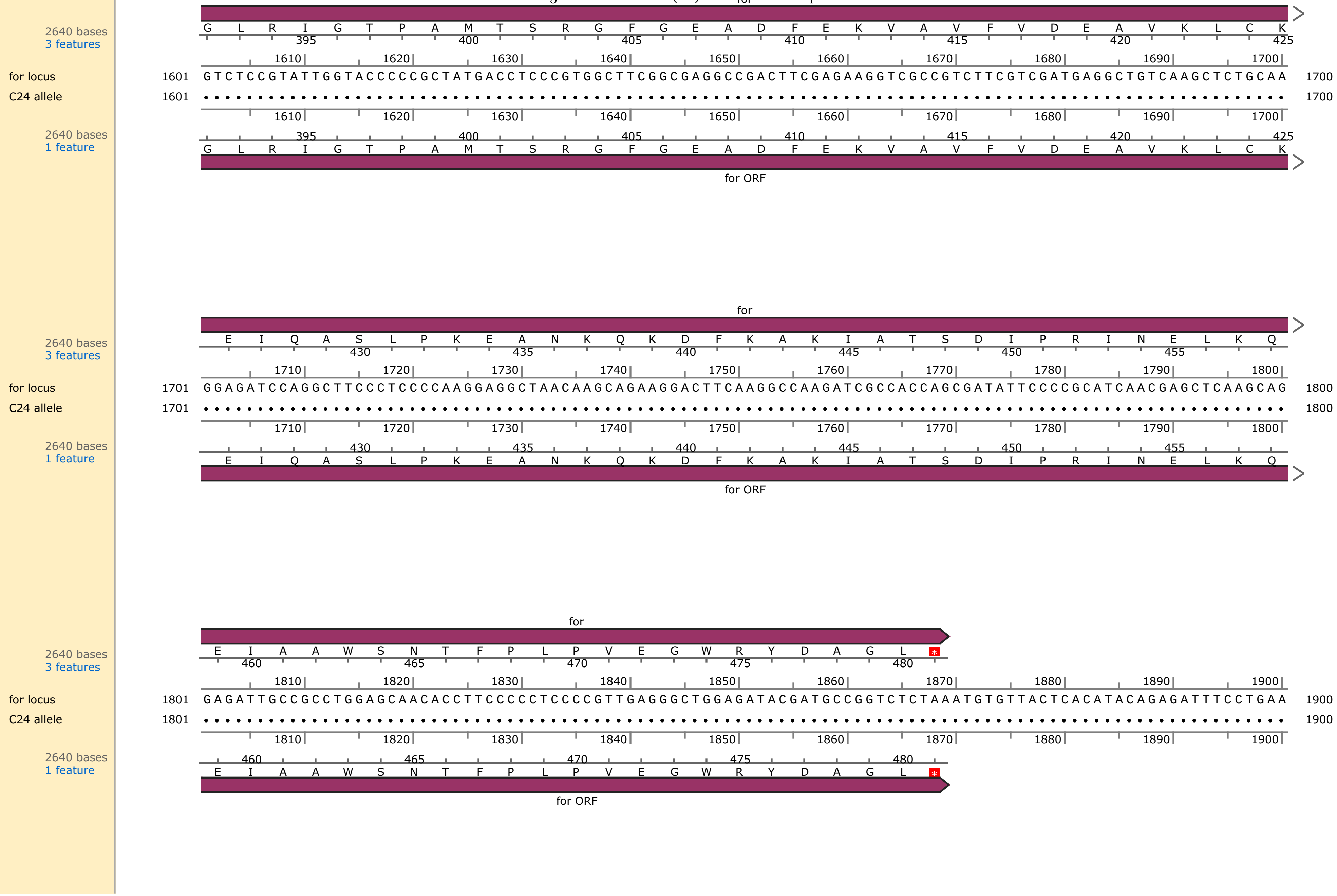
for
2640 bases
3 features
G L R I G T P A M T S R G F G E A D F E K V A V F V D E A V K L C K
395 400 405 410 415 420 425
for locus 1601 GTCTCCGTATTGGTACCCCGCTATGACCTCCCGTGGCTTCGGCGAGGCCGACTTCGAGAAGGTCGCCGTCTTCGTCGATGAGGCTGTCAAGCTCTGCAA 1700
C24 allele 1601 1700
2640 bases
1 feature
for ORF
for
2640 bases
3 features
E I Q A S L P K E A N K Q K D F K A K I A T S D I P R I N E L K Q
430 435 440 445 450 455
for locus 1701 GGAGATCCAGGCTTCCCTCCCCAAGGAGGCTAACAAGCAGAAGGACTTCAAGGCCAAGATCGCCACCAGCGATATTCCCCGCATCAACGAGCTCAAGCAG 1800
C24 allele 1701 1800
2640 bases
1 feature
for ORF
for
2640 bases
3 features
E I A A W S N T F P L P V E G W R Y D A G L *
460 465 470 475 480
for locus 1801 GAGATTGCCGCCTGGAGCAACACCTTCCCCCTCCCCGTTGAGGGCTGGAGATACGATGCCGGTCTCTAAATGTGTTACTCACATACAGAGATTTCCTGAA 1900
C24 allele 1801 1900
2640 bases
1 feature
for ORF

2640 bases
3 features

1910 1920 1930 1940 1950 1960 1970 1980 1990 2000

for locus 1901 TGTTTTGCGTGGTAATGAGGATATACGGTATATAAGCGTTTGGGGCATTTTTCAACAGCGATGCCAATGGGTCGCGGATATGTTTTTCAACTTTGCATGA 2000

C24 allele 1901 2000

1910 1920 1930 1940 1950 1960 1970 1980 1990 2000

2640 bases
1 feature

2640 bases
3 features

2010 2020 2030 2040 2050 2060 2070 2080 2090 2100

for locus 2001 GACCCGGGATGAAGACTTGGGGAAAACTAAAAACAGGGTGGTTTAAAAGGGTCACTGGATTTACGATCTCAACGATGGATTAGGTTACGGGGAAAAAAAA 2100

C24 allele 2001 2100

2010 2020 2030 2040 2050 2060 2070 2080 2090 2100

2640 bases
1 feature

2640 bases
3 features

2110 2120 2130 2140 2150 2160 2170 2180 2190 2200

for locus 2101 GTCACTGGGAGTCGGAAAAAAATTCACCATATTGCATGCATTTCTAAAGGGGCGTTGCAATTTGGTACTTGGTCCTCAACATGATCATTTTTCAGGACTG 2200

C24 allele 2101 2200

2110 2120 2130 2140 2150 2160 2170 2180 2190 2200

2640 bases
1 feature

2640 bases
3 features

2210 2220 2230 2240 2250 2260 2270 2280 2290 2300

for locus 2201 CGGAGTTCGGTGGGGATAACTAGTTGTTTTAGACGACTGCTTCAACGCGGTTGGGGGTCACAAGTTTTGTACCCTGGATCATAATAGACGCAACTATTCT 2300

C24 allele 2201 2300

2210 2220 2230 2240 2250 2260 2270 2280 2290 2300

2640 bases
1 feature

2640 bases
3 features

2310 2320 2330 2340 2350 2360 2370 2380 2390 2400

for locus 2301 TGCGGCAAACTGAGGACGATGGCCCATGTTGTACATAGGTCGGGTTCGTGGTTGTATATGTGTGGATGATGTCTGTGCTCATGAGGGTATGCGTTACGGT 2400

C24 allele 2301 2400

2310 2320 2330 2340 2350 2360 2370 2380 2390 2400

2640 bases
1 feature

DOI: 10.4148/1941-4765.2185

```
2640 bases
3 features
                2410      2420      2430      2440      2450      2460      2470      2480      2490      2500
for locus  2401 ATGATGATAAAAAGTCCGGACACGCTTATACCAAAAACCAGGCCCAGCGAATGTCCAAACCTTTTTCCACACCACCTGGCATGTTGGGGCTTTTGCGATG 2500
C24 allele 2401 .................................................................................................... 2500
                2410      2420      2430      2440      2450      2460      2470      2480      2490      2500
2640 bases
1 feature

2640 bases
3 features
                2510      2520      2530      2540      2550      2560      2570      2580      2590      2600
for locus  2501 ATTCCTTCTGATGTCACGTTGCTAAGGGATACTAGATGGGATTAGATACATGGGATGGATAGATCTACCCGGCTTGCTACCTTTACATACGTATTTGACT 2600
C24 allele 2501 .................................................................................................... 2600
                2510      2520      2530      2540      2550      2560      2570      2580      2590      2600
2640 bases
1 feature

2640 bases
3 features
                2610      2620      2630      2640
for locus  2601 TAGAGATAATTAGGTTGAAGAAATCTGTGCTGGTCGGGAT 2640
C24 allele 2601 ........................................ 2640
                2610      2620      2630      2640
2640 bases
1 feature
```
